# Potential of extracellular space for tissue regeneration in dentistry

**DOI:** 10.3389/fphys.2022.1034603

**Published:** 2022-11-17

**Authors:** Anahid A. Birjandi, Paul Sharpe

**Affiliations:** Centre for Craniofacial and Regenerative Biology, Faculty of Dentistry, Oral and Craniofacial Sciences, Kings College London, London, United Kingdom

**Keywords:** regenerative dentistry, extracellular space, secretome, extracellular vesicles, regeneration

## Abstract

With the proven relationship between oral and general health and the growing aging population, it is pivotal to provide accessible therapeutic approaches to regenerate oral tissues and restore clinical function. However, despite sharing many core concepts with medicine, dentistry has fallen behind the progress in precision medicine and regenerative treatments. Stem cell therapies are a promising avenue for tissue regeneration, however, ethical, safety and cost issues may limit their clinical use. With the significance of paracrine signalling in stem cell and tissue regeneration, extracellular space comprising of the cell secretome, and the extracellular matrix can serve as a potent source for tissue regeneration. Extravesicles are secreted and naturally occurring vesicles with biologically active cargo that can be harvested from the extracellular space. These vesicles have shown great potential as disease biomarkers and can be used in regenerative medicine. As a cell free therapy, secretome and extracellular vesicles can be stored and transferred easily and pose less ethical and safety risks in clinical application. Since there are currently many reviews on the secretome and the biogenesis, characterization and function of extracellular vesicles, here we look at the therapeutic potential of extracellular space to drive oral tissue regeneration and the current state of the field in comparison to regenerative medicine.

## Introduction

Medicine is rapidly advancing towards precision therapy, using the latest advancements in biotechnology to provide patient-tailored treatments. In dentistry the emphasis has been placed more on enhancing the quality and aesthetics of dental fillings or impression materials and use of digital technology for diagnosis and rapid chair side treatments. With the growing aging population and the predicted increased number of people aged 65 and over by 2050, the need for a more accessible, efficient and targeted dental treatment is more palpable to regenerate dental tissues and restore clinical function (UN Population Division, 2019).

Functional tooth is comprised of innervated and vascularized dental pulp encased by dentin, cementum, and the harder enamel as the external protective layer. Periodontal or tooth supporting tissue consists of periodontal ligament, gingiva and alveolar bone. Dental caries and trauma often require restoration of the tooth by placement of filling material in the crown or endodontic therapy, to remove and replace dental pulp. Inflammation of tooth supporting tissue results in periodontitis, loss of alveolar bone, tooth mobility and ultimately tooth loss. Conventional treatment replaces the missing tooth with removable dentures, fixed restoration such as crown, bridges, or placement of an implant. All of which aim to replace a biological tissue with metal prosthesis.

Regenerative dentistry aims to replace conventional dental treatments with biologically driven approaches to induce repair of the diseased tissue, reduce inflammation and eliminate scarring. These therapies aim to be minimally invasive and can involve local delivery of active molecules into the region of interest to stimulate tissue regeneration. Regenerative approaches may be relatively easy for some dental tissues such as dental pulp since dental pulp cells can be grown and maintained in culture and subsequently applied in regenerative endodontics (Brizuela et al., 2020; Matoug-Elwerfelli et al., 2020). Similarly, conventional filling materials can be replaced by biological alternatives that stimulate stem cells and promote tissue regeneration in dentin or enamel (Neves et al., 2017; Elsharkawy et al., 2018; Birjandi et al., 2020). Treatment of edentulous patients can ultimately be achieved by replacing the whole tooth organ with bioengineered tooth or transplantation of constructed embryonic tooth primordia into the oral cavity (Yelick and Sharpe, 2019). It is however necessary to consider avenues other than cell-based approaches for regeneration of these tissues.

In regenerative medicine, Bioprinting, iPSCs and patient derived stem cells can be used for tissue repair. Similarly, Nanoparticle and Nanoclay technology are applied for drug delivery (Dutta and Dutta, 2012; Balint et al., 2013; Zhang and Zhang, 2015; Coyle et al., 2016; Dogan et al., 2017; Im, 2018; Hoveizi et al., 2019; Xie et al., 2022). New tools have been developed for minimally invasive surgical approaches, but further improvements are still required for regenerative therapies to become effective for all major diseases. Scaling up the size and speed of manufacturing, reproductivity and reliability for clinical translation are some of the matters that need tackling in regenerative medicine. Additionally, more advanced assays should be developed to better understand the cellular behaviour in these treatments and accurately predict the performance of novel therapeutics *in vivo* (Tucker et al., 2016). Regenerative dentistry faces additional challenges due to lack of emphasis for biologically driven therapeutics in clinics, strong marketing aspect of the profession and lack of support for clinical trials of regenerative therapies should they pass the preclinical tests.

## Regenerative potential of extracellular space

Much work has been done on the development of new materials to recapitulate cellular function and promote tissue regeneration. Autologous or allogenic cell therapies can contribute to tissue regeneration, but the low proliferative potential of differentiated cells and their limited availability has restricted their use. Therefore, although stem cell therapy is a promising avenue for tissue regeneration, issues such as immune compatibility, risk of infection and tumorigenicity have prevented these treatments from becoming the mainstream regenerative approach (Maguire, 2013; Eggenhofer et al., 2014; Jiang et al., 2014; Laurencin and Nair, 2014; Turinetto et al., 2016; Menasché, 2018; Arnold et al., 2019; Zhou et al., 2019; Laurencin and Daneshmandi, 2020; Matai et al., 2020).

For years the focus of developmental biology, the base of regenerative biology, has been on the contents of the cells and the role of intracellular events in cellular behaviour and function. Today, there is an increasing appreciation of the extracellular space as a force to drive cellular function, stemness, homeostasis, healing, survival and motility (Naba et al., 2016; Laura, 2018). Furthermore, it is established that the paracrine effect of stem cells plays a major role in tissue repair and regeneration. This effect is through the extracellular space which is composed of the ECM and the cell secretome.

The ECM is a network of proteoglycans, glycosaminoglycans and proteins arranged in a 3D architecture that provides structural properties and signalling cues whilst serving as the microenvironment that diffuses nutrients. Therefore, ECM and ECM-derived biomaterials are extensively studied for their potential in tissue regeneration and as delivery vehicle for stem cell treatments (Capella-Monsonís et al., 2020). For example, Melatonin-loaded hydrogels show favourable viscoelastic properties and promote accelerated and enhanced quality of bone formation in regeneration of furcation defect in dogs (Abdelrasoul et al., 2022). Specifically, extracellular matrix derived from dental pulp stem cells can promote mineralization of human gingival fibroblasts (Nowwarote et al., 2022). Dual ECM scaffolds derived from pulp and endothelial ECM promote attachments of mesenchymal stem cells and subsequently odontogenic differentiation. Subcutaneous implantation of these scaffolds into tooth root slice model *in vivo* results in odontogenic differentiation of dental pulp and bone marrow derived MSC (Huang et al., 2018). ECMs are also used in disease modelling and cancer. Indeed, cell derived ECMs are great tools to study cancer microenvironment as they provide a more physiologically relevant phenotype of cancer cells in comparison to conventional 2D and 3D culture models (Serebriiskii et al., 2008; Eberle et al., 2011; Kaukonen et al., 2017; Hoshiba, 2018). This interesting application of ECM has not been tested extensively in oral dysplasia or cancer models such as squamous cell carcinoma. Further understanding and correlation of the composition and biological function of ECM is essential in regenerative approaches of complex oral tissues.

Secretome is composed of the factors that are actively or passively released by the cells. This ranges from cytokines, chemokines, growth factors, lipids to free nucleic acids and extracellular vesicles. The secretome has been shown to have various roles in tissue repair, angiogenesis and immunoregulation (Xia et al., 2019; Willis et al., 2020; Damayanti et al., 2021). Secretome can be easily harvested, freeze dried and transported. As a cell free approach, secretome therapy does not induce the adverse effects of cell therapies such as tumorgenicity and host rejection (Song et al., 2019; Mocchi et al., 2021). An important and biologically active component of the secretome are Extracellular vesicles. Extracellular vesicles (EV) are naturally occurring vesicles with biological cargo that mediate cell-cell communication. The field of extracellular vesicles is constantly evolving with the identification of new groups of nanoparticles based on their size, function and cargo such as exosomes, ectosomes, exomeres, supremeres and migrasomes (Raposo and Stoorvogel, 2013; Cocucci and Meldolesi, 2015; Zhang et al., 2018; Zhang et al., 2021). Some of the regenerative cargo within native, unmodified extracellular vesicles (EVs) are mRNA, miRNA, proteins. Therefore, EVs can be used as biomarkers of different diseases as well as cancer. A recent work has shown that EVs can contribute to cancer aggressiveness (Nigri et al., 2022). Equally EVs can modulate activity of immune cells and remodelling of ECM and subsequently contribute to tissue repair (Silva et al., 2017). As an example extracellular vesicles protect the bone and cartilage from degradation and promote bone regeneration (Cosenza et al., 2017; Jia et al., 2019). Bone marrow stem cells (BMSCs) treated with EVs promote healing of calvarial defect in mice (Li et al., 2020). Similarly, EVs derived from human Placenta MSC and human BMSC promote myelin regeneration and functional recovery (Hu et al., 2016; Clark et al., 2019). EVs can be traced back to their cells of origin and exhibit similar or even enhanced *in vivo* performance in comparison to their parent cells (Larssen et al., 2017; Chen and Guo, 2020). Extracellular vesicles derived from BMSCs promote liver regeneration with the same efficiency as BMSCs in a mouse model of hepatic ischemia (Anger et al., 2019). However, In a rat model of myocardial infarction, MSC derived EVs performed better than their MSC (Shao et al., 2017). Similarly iPSC-derived cardiomyocyte EVs have enhanced performance in comparison to cell injection in mice (El Harane et al., 2018). The regenerative effect of EVs can even be modified by altering the culture condition of their parent cells (Murphy et al., 2019; Ramasubramanian et al., 2019). Hypoxic condition results in increased biological activity of the cell and subsequently increased regenerative potential of their secreted EV (Hu and Li, 2018). Culturing osteoblasts in the presence of mineralizing supplements results in EVs that are capable of mineralization in MSC cultures. Similarly, priming MSCs derived from adipose tissue with TNF-a results in EV secretion and osteogenic differentiation of human primary osteoblasts (Davies et al., 2017; Lu et al., 2017; Lee and Kim, 2021; Liu and Holmes, 2021; Nagelkerke et al., 2021). EVs can also be bioengineered and enriched with favourable factors such as drugs and growth factors to serve as targeted therapies (Kim et al., 2022). All of these demonstrate the huge potential of EVs for tissue regeneration.

EVs can be derived from dental mesenchymal stem cells (DMSC). These cells have the advantage of enhanced regenerative ability and good accessibility. DMSC can be harvested from multiple sites in the oral cavity such as Dental pulp stem cells, exfoliated deciduous tooth stem cells, apical dental papilla stem cells, periodontal ligament stem cells, dental follicle stem cells, gingiva derived mesenchymal stem cells and tooth germ progenitor cells (Chouaib et al., 2022). Similar to their parent cells, conditioned medium or secretome form dental mesenchymal stem cells (DMSCs) exhibits great regenerative potential and possess greater amount of metabolic and proliferative associated proteins, neutrophils and chemokines in comparison to non-dental conditioned medium (El Moshy et al., 2020). Interestingly, DMSCs has been shown to promote tissue regeneration in liver (Hirata et al., 2016; Matsushita et al., 2017), and in many pathological conditions such as diabetes and neurological disorders (Izumoto-Akita et al., 2015; Jarmalavičiūtė et al., 2015; Mita et al., 2015; Ahmed et al., 2016; Asadi-Golshan et al., 2018; Wang et al., 2019; Chen et al., 2020), Cardiac and pulmonary lesions (Wakayama et al., 2015; Yamaguchi et al., 2015) and alveolar bone defects (Omori et al., 2015; de Cara et al., 2019; Hiraki et al., 2020; Qiu et al., 2020). Despite these discoveries, there are limited number of studies on the characterization and biological function of dental mesenchymal stem cell derived secretome or exosomes in comparison to other sources of EVs in regenerative medicine. Additionally, not all the studies using DMSC secretome have characterized the stemness and differentiation capacities of the EVs and their cell of origin in full detail. Additionally, There is also a degree of variability in factors affecting the cells of origin such as passage number, sorting of the cell and period of conditioning that subsequently affects the secretome and its biological function across different studies (Chouaib et al., 2022).

## Challenges in secretome therapy for tissue regeneration

The promising results of EVs and their benefit over cell-based therapeutic approaches has resulted in them being tested in clinical settings and their clinical safety being approved (Escudier et al., 2005). As a cell free approach, secretome and extracellular vesicles do not present the ethical and safety issues associated with conventional cell therapies such as immune compatibility, risk of infection and tumorigenicity. Additionally, they can be easily harvested, stored, transferred and applied. Administration of autologous EV minimises the risk of pathogen transmission and immunological intolerances and this is can be a great advantage in assessment of EV function in clinical setting and phase I or II trials (Beer et al., 2017).

Despite these advantages, the role of EVs as sole therapy still requires further investigation. Identification of the active molecule that exerts the biological function in EVs is extremely challenging. Sensitivity of mass spectrometers should be able to cover the range of low abundance proteins over highly abundant proteins in the secretome and EVs to better characterize them (Brown et al., 2012). The purify of final EV population and their heterogeneous nature is equally important. This is because the contents and therefore biological activity or regenerative capacity of EVs may vary depending on the cell type, experimental setup and purification method. Variation in the existing methods of purification, characterization and storage of cells, their secretome and EVs are some of the other challenges in the field although many of these are currently being addressed.

To bypass some of the limitations of natural occurring EVs and avoid some of the mentioned inconsistencies, EVs can be bioengineered and enriched with favourable factors resulting in enhanced yield and better isolation but the full impact of these modifications on target cells needs to be investigated in detail (Kim et al., 2022). In clinical translation, it is yet to be determined which donor cell source can provide the most potent EV subtypes and which factors influence this (Beer et al., 2017). Mass production of EVs is also challenging. Even the golden standard of EV purification with ultracentrifuge has its own setbacks such as availability of the equipment in all labs, potential of co-isolation of non-vesicle macromolecules with EV and EV aggregation. Ultracentrifugation is also time consuming. However commercial EV purification kits that are currently available are not yet suitable for clinical application. It is therefore important to establish the manufacturing workflow for suitable EV purification according to good manufacturing practice (GMP) and enhance their storage condition and accessibility for clinical application. EV therapeutics are currently categorized as biologicals with active substance but it is still not clear if a step for pathogen reduction would be implemented in mass production of EV and if so, how that step affects the morphology and integrity of EV and subsequently the interaction with target cells and biological activity (Beer et al., 2017).

Majority of currently running EV clinical trials are aimed to monitor circulating EV as biomarkers, disease progression and cancer diagnosis and very few are addressing tissue repair and regeneration. With the proven link between the oral and general health, designing preclinical studies and clinical trials that aim to improve oral health using regenerative approaches is important. The great advantage of cell free approaches over cell-based therapies, makes secretome and EV a very attractive tool for regenerative treatment. Much work must be done to uncover the full potential of secretomics in oral health and to then develop standards for reproducibility, clinical transition and accessibility for public.
